# Recurrent Radial Head Dislocation

**Published:** 1991-06

**Authors:** Barbara Michelmann

**Affiliations:** Medicine Student Medizinische Hocheschule Hannover, Germany

## Abstract

Radial head subluxation is a common injury in children. It has been described as nurse's elbow, radial head subluxation (RHS) and "pulled elbow". There may be a recurrence, however this is the first time that it has been reported to recur three times.


### Recurrent Radial Head Subluxation in a 3 Year Old Child — Case Report

**Barbara Michelmann, Medicine Student**
Medizinische Hocheschule Hannover, Germany

### SUMMARY
Radial head subluxation is a common injury in children. It has been described as nurse's elbow, radial head subluxation (RHS) and "pulled elbow". There may be a recurrence, however this is the first time that it has been reported to recur three times.

### INTRODUCTION
Radial head subluxation (RHS), a condition also known as "pulled elbow" was first described by Hugh Owen-Thomas in 1883. It is a common paediatric orthopaedic injury, diagnosis of which is generally based on the classic history of a longitudinal pull on the pronated and extended forearm[1]. In this pronated and extended position the narrowest diameter of the radial head is in the anteroposterior plane and as the radius moves distally a transverse tear is produced in the thin distal attachment of the annular ligament. This then becomes interposed between the surfaces of the radial head and the capitellum when traction is discontinued[2]. The child characteristically presents with the affected limb held slightly flexed and pronated and supports the forearm with the other hand[5]. Reduction by manipulative supination results in instant symptomatic relief and is associated with a palpable and/or audible click. The radiographs are usually reported to be normal[1,6], for the radial head moves back into its normal position once the initial traumatic traction stops[2].

RHS is most common in toddlers aged 1–3, in girls more than boys[4], and in the left elbow more than the right[3]. Irreducibility is rare, occurring only in older children[2].

### THE CASE REPORT
A 3 year old girl was seen on December 9th 1990 at Weston-super-Mare General Hospital Accident and Emergency Department having sustained an injury to her left elbow less than 2 hours previously. The father stated that while holding on to a tablecloth, which he himself pulled on at the same time, she suddenly screamed. She would not use the arm and held the limb loosely by her side supported by the other hand. On examination she would not actively move the elbow. Passive movement was full with the exception of supination which was resisted and caused her to scream out. Palpation showed slight tenderness over the anterior-lateral aspect of the radial head. A diagnosis based entirely on the history and clinical findings, of "pulled elbow" was then made. Manipulative supination produced an audible click and resulted in normal use of the arm within 10 minutes. Active forearm supination could be performed without distress. A crepe bandage was applied.

The father stated that a similar condition had occurred thrice previously during the previous 12 months, the last episode being just 2 weeks prior to this admission, when her 8 year old cousin had picked her up. The parents had been advised how to handle the child after the first instance and were obviously distressed over the recurrence.

### DISCUSSION
It is unusual for RHS to occur more than twice on the same side[3].

This is the first description of 4 subluxations. Recurrent RHS and repeated manipulative supination within a short time may result in lateral instability as well as irreducibility due to repeated trauma to the thin annular ligament. Consequently in older children, on rare occasions, a surgical tightening of the annular ligament may become necessary. This should be carried out after growth has ceased[8].

I would like to emphasise that even though this is considered a minor paediatric injury, the distress to the injured child and its family can be immense and that great care should be taken to inform the family about the mechanisms of the injury and the possible causes[5]. Furthermore it is important to protect the child from other children by putting the affected limb into some form of non-restricting visible bandage for between 2 and 4 weeks after injury to reduce the possibility of a further recurrence.

### REFERENCES
1. WOO, C. (1987) Traumatic radial head subluxation in young children: A case report and literature review. *J. Manipulative Physiol. Ther.* **10**(4), 191–200.
2. SALTER, R.B. *ET AL.* (1971) Anatomic investigations of the mechanism of injury and pathological anatomy of "pulled elbow" in young children, *Clin. Orthop.* **77**, 134–143.
3. QUAN, L. *et al.* (1985) The epidemilology and treatment of radial head subluxation. *Am. J. Dis. Child.* **139**(12), 1194–1197.
4. AMIR, D. *ET AL.* (1990) Pulled elbow and hypermobility of joints. *Clin. Orthop.* **257**, 94–99.
5. SACCHETTI, A. *et al.* (1990) Nonclassic history in children with radial head subluxation. *J. Emerg. Med.* **8**(2).
6. SYNDER, H.S. (1990) Radiographic changes with radial head subluxation in children. *J. Emerg. Med.* **8**, 265–269.
7. THOMAS, H.O. (1883) The principles of treatment of diseased joints p.77, London Lewis.
8. WIRTH, C.J. (1988) Sekundaten im Ellenbogengelenksbereich. *Orthopade.* **17**(4), 353–358.

### ACKNOWLEDGEMENTS
I am indebted to Mr P.H. Roberts, Consultant Orthopaedic Surgeon at Weston General Hospital who has made this paper possible and taught me during my attachment with the department.

### ABOUT THE AUTHOR
Miss Michelmann is a 6th year medical student from the Medical University of Hannover, Germany. She is at present at Weston-Super-Mare General Hospital for an elective period of 11 months before her final year, to gain practical experience in various fields of medicine.
P.H. Roberts.

